# Accurate, Fully-Automated NMR Spectral Profiling for Metabolomics

**DOI:** 10.1371/journal.pone.0124219

**Published:** 2015-05-27

**Authors:** Siamak Ravanbakhsh, Philip Liu, Trent C. Bjordahl, Rupasri Mandal, Jason R. Grant, Michael Wilson, Roman Eisner, Igor Sinelnikov, Xiaoyu Hu, Claudio Luchinat, Russell Greiner, David S. Wishart

**Affiliations:** 1 Department of Computing Science, University of Alberta, Edmonton, AB, Canada; 2 Alberta Innovates Center for Machine Learning, Edmonton, AB, Canada; 3 Department of Biological Sciences, University of Alberta, Edmonton, AB, Canada; 4 Fiorgen Foundation, 50019 Sesto Fiorentino, Florence, Italy; 5 Centro Risonanze Magnetiche, University of Florence, Florence, Italy; 6 National Research Council, National Institute for Nanotechnology, Edmonton, AB, Canada; Instituto de Investigación Sanitaria INCLIVA, SPAIN

## Abstract

Many diseases cause significant changes to the concentrations of small molecules (a.k.a. metabolites) that appear in a person’s biofluids, which means such diseases can often be readily detected from a person’s “metabolic profile"—i.e., the list of concentrations of those metabolites. This information can be extracted from a biofluids Nuclear Magnetic Resonance (NMR) spectrum. However, due to its complexity, NMR spectral profiling has remained manual, resulting in slow, expensive and error-prone procedures that have hindered clinical and industrial adoption of metabolomics via NMR. This paper presents a system, BAYESIL, which can quickly, accurately, and autonomously produce a person’s metabolic profile. Given a 1D ^1^ H NMR spectrum of a complex biofluid (specifically serum or cerebrospinal fluid), BAYESIL can automatically determine the metabolic profile. This requires first performing several spectral processing steps, then matching the resulting spectrum against a reference compound library, which contains the “signatures” of each relevant metabolite. BAYESIL views spectral matching as an inference problem within a probabilistic graphical model that rapidly approximates the most probable metabolic profile. Our extensive studies on a diverse set of complex mixtures including real biological samples (serum and CSF), defined mixtures and realistic computer generated spectra; involving > 50 compounds, show that BAYESIL can autonomously find the concentration of NMR-detectable metabolites accurately (~ 90% correct identification and ~ 10% quantification error), in less than 5 minutes on a single CPU. These results demonstrate that BAYESIL is the first fully-automatic publicly-accessible system that provides quantitative NMR spectral profiling effectively—with an accuracy on these biofluids that meets or exceeds the performance of trained experts. We anticipate this tool will usher in high-throughput metabolomics and enable a wealth of new applications of NMR in clinical settings. BAYESIL is accessible at http://www.bayesil.ca.

## Introduction

Metabolomics is a relatively new branch of “omics” science that focuses on the system-wide characterization of small molecule metabolites and small molecule metabolism [1, 2]. Metabolomics is often viewed as complementary to the other “omics” fields as it provides information about both an organism’s phenotype and its environment [3]. Because metabolomics provides a unique window on gene-environment interactions, it is playing an increasingly important role in many quantitative phenotyping and functional genomics studies [4–8]. It is also finding more applications in disease diagnosis, biomarker discovery and drug development/discovery [9–12].

This rapid growth in interest and excitement surrounding metabolomics is also revealing its “Achilles heel”: Unlike proteomics, genomics or transcriptomics, which are *high-throughput* sciences, metabolomics is a relatively *low-throughput* science. Compared to genomics, where it is now possible to automatically characterize 1000s of genes, 100s of thousands of transcripts and millions of SNPs in mere minutes, metabolomics only allows users to identify and measure a few dozen metabolites after many hours of manual effort. In other words, *metabolomics is not yet automated*.

This problem may stem from the history of metabolomics, as its analytical techniques, such as nmr spectroscopy, gas-chromatography-mass spectrometry (GC-MS) and liquid chromatography-mass spectrometry (LC-MS), were originally developed for identifying and quantifying *pure* compounds, not complex mixtures. Because most biological samples contain hundreds of metabolites, the resulting NMR, HPLC or LC-MS spectra usually contain hundreds or even thousands of peaks. The challenge in metabolomics, therefore, is to identify the mixture of compounds that produced this forest of peaks. This compound identification process, called *spectral profiling*, involves fitting the mixture spectrum to a set of individual pure reference spectra obtained from known compounds [13–15]. If done correctly, the fitting process yields not only the identity of the compounds, but also the concentration of those compounds. Therefore, the end result of a successful spectral profiling study is a table of metabolite names and their absolute or relative concentrations. Because spectral profiling is such a complex pattern recognition problem, it is often best done by a trained expert. However, this reliance on manual data analysis by a human expert is problematic, as it is slow and leads to inconsistent results, operator errors and reduced levels of reproducibility [16].

The automation bottleneck in metabolomics is widely recognized, and has led to a number of efforts to accelerate or automate compound identification and/or quantification in LC-MS, in GC-MS and in NMR spectroscopy. Some of the most active efforts in (semi)automated compound identification and quantification have been in NMR-based metabolomics. In particular, several software packages have been developed that support semi-automatic NMR spectral profiling of 1D and 2D ^1^ H NMR spectra, including some commercial packages [17–19]. However, these packages either require manual fitting or manual spectral processing, or a bit of both (see S1 Appendix for a comprehensive list of NMR software packages and their limitations.) The need for such manual interventions leads to a number of issues, including slower throughput, operator fatigue and associated operator errors, the need for highly trained and dedicated experts, the requirement of two or more spectral assessments for quality assessment and control purposes, and inconsistent results between individuals, between labs or over different time periods [13, 16].

It would be better to have a software system that can automatically perform both spectral processing and spectral profiling, be able to analyze complex mixtures quickly and accurately, and be able to produce reliable compound concentrations. Here we describe such a system, called BAYESIL, the first system that supports fully automated and fully quantitative NMR-based metabolomics of complex mixtures. In this paper we demonstrate that our system can effectively profile human serum and CSF samples, each containing ∼ 50 compounds. Our lab is currently implementing extensions to other biofluids or extracts containing even more compounds.

## Materials and methods

BAYESIL performs fully automated spectral processing and spectral profiling for 1D ^1^ H NMR spectra collected on standard (*i.e.*, either Agilent/Varian or Bruker) instruments, at several different frequencies. In particular, it uses a variety of intelligent phasing and baseline correction methods to automatically process raw 1D NMR spectra—*a.k.a*. free induction decay (FID). During spectral deconvolution, BAYESIL divides the spectrum into small blocks and represents the sparse dependencies between these blocks using a “probabilistic graphical model”. It then performs approximate inference over this model as a surrogate for spectral profiling, yielding the most probable metabolic profile. Here, we briefly describe BAYESIL’s spectral processing algorithms, the principles and rationale behind BAYESIL’s spectral profiling method and the construction of BAYESIL’s spectral library.

### Spectral processing in BAYESIL

**Fig 1:**
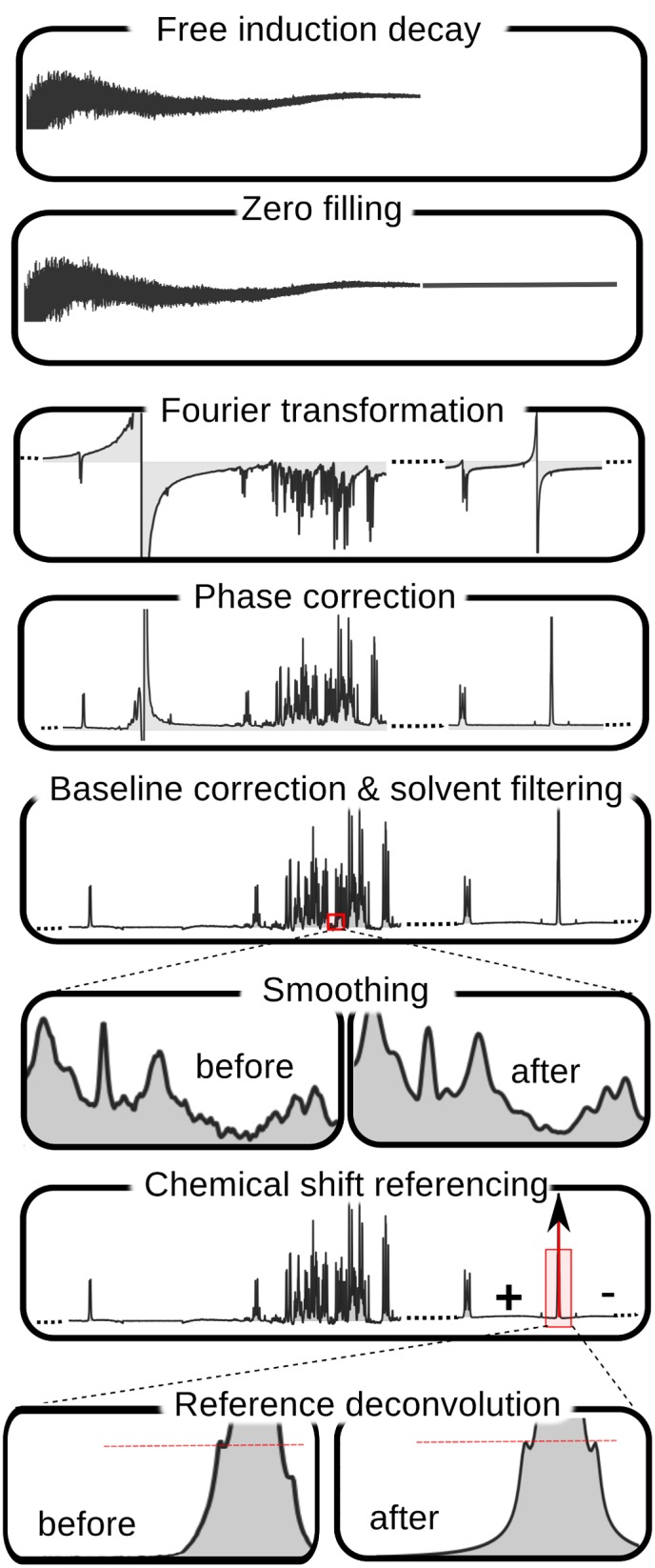
Spectral processing steps in Bayesil. Reference deconvolution and smoothing are optional. After baseline correction, Bayesil may go back to phase correction to re-adjust the phasing. For this, the imaginary part of the spectrum is reconstructed using Hilbert transformation (not shown).

Successful NMR spectral profiling depends critically on the quality and uniformity of the starting NMR spectrum. Unfortunately, most spectral processing functions (*i.e.*, phasing, baseline correction, solvent filtering, chemical shift referencing) are left to the user. Given the complexity and large number of variables, values and filters that can be used, many view spectral processing more as an art, rather than a science. Different perspectives or different personal thresholds on what is a “good looking” NMR spectrum can potentially lead to very different results regarding what compounds are identified or which compounds are accurately quantified in a biofluid spectrum. To address this issue, BAYESIL itself performs all of the spectral processing functions (see Fig 1): starting from the raw spectrum, it performs zero-filling, Fourier and Hilbert transformation, phasing, baseline correction, smoothing, chemical shift referencing and reference deconvolution. Automating this process ensures reproducibility, consistency and uniformity of the input data prior to spectral profiling. Here we briefly sketch some of the more challenging steps in this process.

*Phasing* involves maximizing the symmetry of the peaks by reducing zero-order and first-order phase mismatch. Zero-order phase mismatch is a sign of the difference between the reference phase and the receiver phase and is independent of frequency. The first-order phase mismatch can be a result of the time-delay between excitation and detection, flip-angle variation and the filter that is used to reduce the noise outside of the spectral bandwidth [20]. In addition to using well-known techniques, such as spectral norm minimization [21], BAYESIL uses the cross entropy optimization method [22, 23] to jointly maximize a direct measure of peak symmetry for isolated peaks across the spectrum.

*Baseline correction* involves removing distortions that may arise from hardware artifacts or highly concentrated components of the mixture (*e.g.*, solvent), while keeping the desirable signal intact [24]. This process is often performed in two steps: 1) baseline-detection and 2) modelling. BAYESIL relies on iterative thresholding [25] and estimating the signal-to-noise ratio to detect the baseline points. It uses monotonic cubic Hermite interpolation [26] and Whittaker smoothing technique for baseline modelling [27].

BAYESIL also provides the options for *smoothing and line-broadening* using Savitzky-Golay [28] and Gaussian filters. However smoothing is mostly cosmetic and it is not essential for spectral profiling. In fact, it may degrade the signal and occasionally remove the the low-amplitude and narrow peaks. Similarly, we found the effect of *reference deconvolution*—which may be used to remove instrumental or experimentally induced distortions of the Lorentzian lineshape [29] – is also mostly cosmetic, and if the distortion around the reference peak has any source other than poor shimming, using reference deconvolution will have an adverse effect on the rest of the NMR spectrum.

### Spectral profiling

NMR spectrum of a compound ℳ is a set of clusters {𝓒_*k*_}, where each cluster 𝓒_*k*_ is set of “Lorentzian” peaks, and each peak is defined by three parameters, corresponding to its height, center and width (at half height). These parameters are constant across different spectra of the same frequency and a *compound library* records this information for various compounds.

However, the spectrum of a pure compound is also associated with two “variables”. The *compound concentration* *ρ*_ℳ_ linearly scales the peak heights—*i.e.*, doubling the concentration results in peaks that are twice as high. Moreover different clusters 𝓒_*k*_ can *shift* within a small window, offsetting the center of all the peaks in the same cluster by some (random) value *δ*_𝓒_. Therefore, having access to a compound library, the concentration *ρ*_ℳ_ and a set of shift variables ***δ***_ℳ_{*δ*_𝓒_∣𝓒 ∈ ℳ} completely define the spectrum of a pure compound.

An NMR spectrum of a *mixture* is essentially a linear combination of the spectra of its compounds: that is, the height at each location is just the sum of the contributions of each compound. This means, given the concentrations of the compounds ***ρ*** = {*ρ*_ℳ_}, and the chemical shifts ***δ*** = ⋃_ℳ_ ***δ***_ℳ_ of the clusters associated with these compounds, we can then “draw” an NMR spectrum for a mixture. The spectral profiling challenge, in general, is the reverse process: Given a set of compounds {ℳ_1_, …, ℳ_*r*_} with associated signatures in a compound library and the observed spectrum, find the “best” combination of concentrations ***ρ*** and shifts ***δ*** to fit that spectrum.

**Fig 2:**
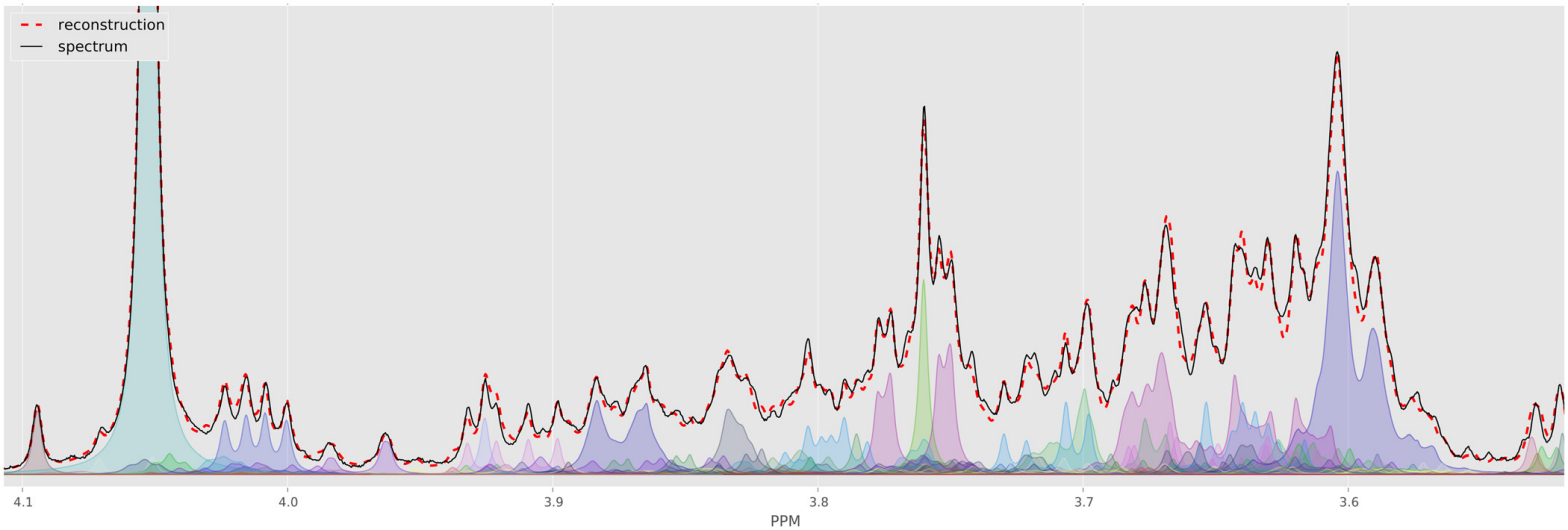
The crowded region (3.5–4.1 ppm) of a computer generated spectrum with 150 compounds (solid black) and the fit produced by Bayesil (dashed red) as well as individual clusters as quantified by Bayesil. Each cluster is free to shift a specified amount, which is at least 0.025 PPM.

This is often quantified using a loss function that measures the difference between the input spectrum and its reconstruction. However, even for a simple loss function (*e.g.*, sum of squared errors), finding the best assignment corresponds to search over a huge space—all possible shifts for each of the clusters, and all possible concentrations over the compounds. This highly nonlinear and high-dimensional optimization problem has been the main challenge in automating NMR spectral profiling and a key innovation of BAYESIL is in efficiently solving this problem. Fig 2 shows part of a spectrum for a complex mixture, and BAYESIL’s solution obtained by minimizing the loss function.

### Factorization and inference

**Fig 3:**
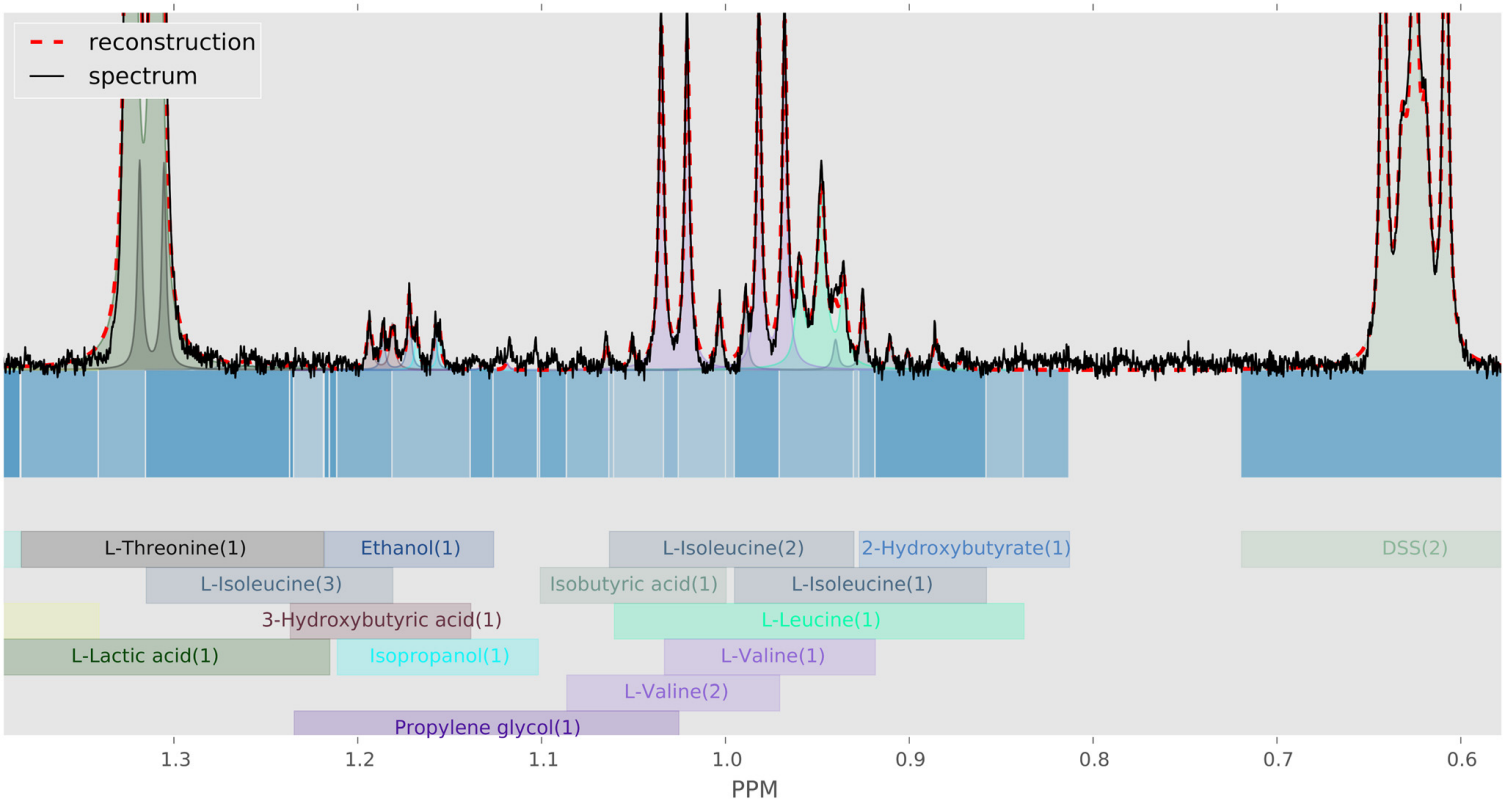
Construction of spectral regions. Partitioning of spectrum 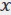 into continuous blocks 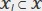. Here each block is shown with a different shade of blue, below the horizontal axis. The domain of influence of each cluster is also indicated with coloured blocks, where each cluster assumes the same colour in reconstruction $\hat{\text{s}}$ of the spectrum (above horizontal axis).

BAYESIL “factors” the spectrum and the loss function into a set of inter-related regions and functions. Two characteristics of the NMR spectra make this factorization possible: 1) each shift is over only a small range (typically a window of ±0.025 PPM); and 2) as the height of a (Lorentzian) peak diminishes quickly from its center, each peak and therefore each cluster can only “influence” a small interval. BAYESIL partition the spectrum into disjoint contiguous regions, such that every point in each region involves exactly the same subset of clusters. Fig 3 shows the division of a part of human serum NMR spectrum into regions; blocks in different shades of blue.

BAYESIL then takes a probabilistic approach using the *Gibbs* distribution [30], such that an *undesirable assignment* to [***δ***,***ρ***] which also has a high loss value, will have a *low probability* ℙ(***ρ***,***δ***). This transformation from a loss function to a probability distribution has its origin in statistical physics where it relates the notion of “energy” to probability, such that low energy states have higher probabilities.

By dividing the NMR spectrum into blocks, this distribution also decomposes over these regions and can be represented using a *probabilistic graphical model*, known as a factor graph [31]. Probabilistic graphical models and in particular factor-graphs are credited with several breakthroughs in different fields; from solutions to the most notorious satisfiability problems [32], to codes that achieve theoretical optimum in communication through noisy channels [33]. In bioinformatic, beside their application in modeling regulatory networks [34] a classic and simple variation of probabilistic graphical models known as hidden Markov model has been used in many applications including sequence alighnment, RNA structural alighnment, folding and annotation, pedagogy trees and protein secondary structure prediction.

**Fig 4:**
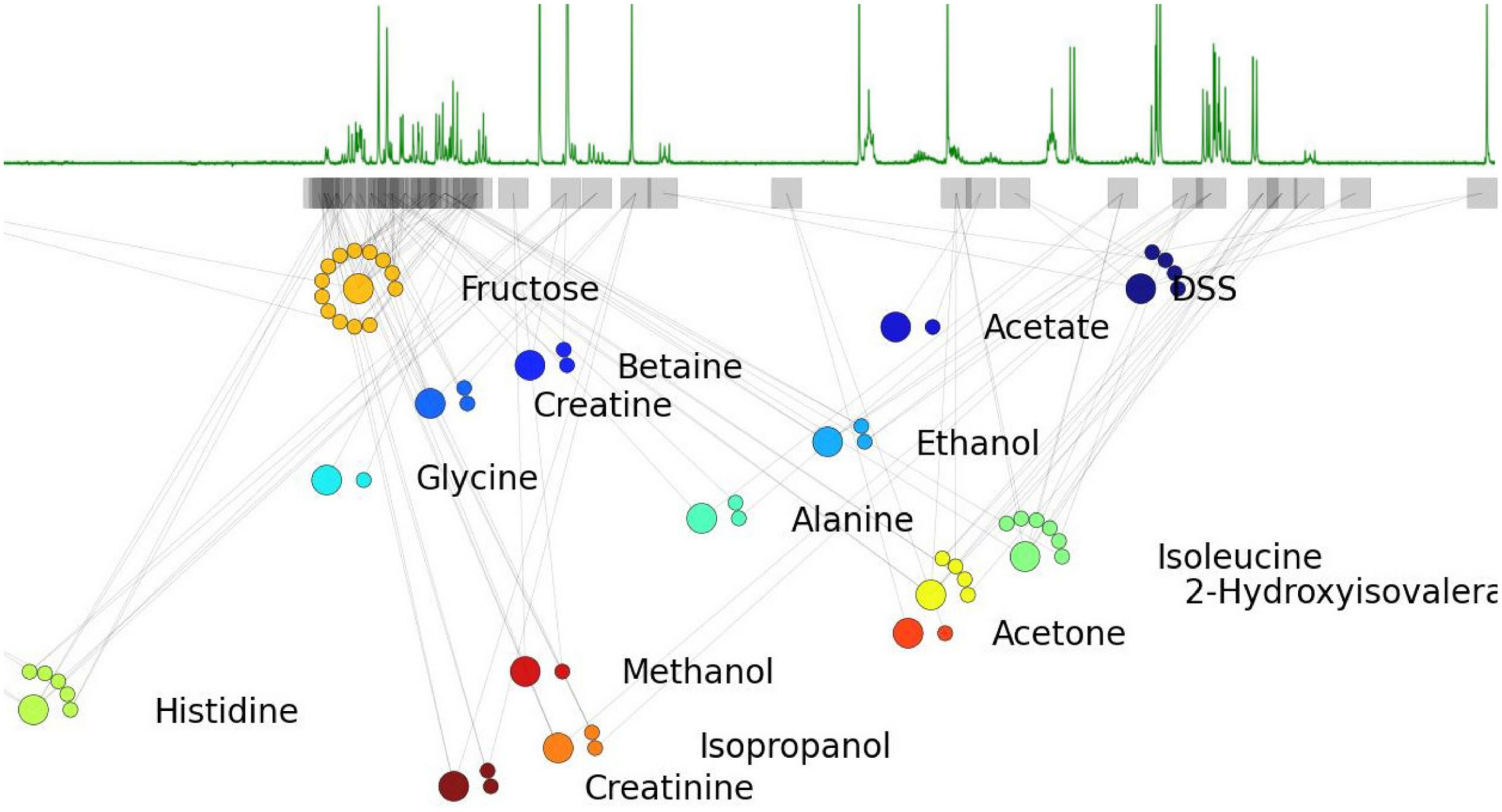
The factor-graph associated with a simple NMR spectrum. The factor-graph for a library of 15 compounds is shown immediately below an associated NMR spectrum. Each factor is represented by a square and each variable using a circle. Concentration (larger circles) and shift variables (smaller circles, beside the associated concentration) corresponding to each compound appear together. The position of each factor ${\text{f}}_{I}$ position in the plot corresponds to the center of the corresponding block 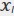.

The point of convergence for these models and methods is decomposition of a probability distribution to a set of interdependent factors, which then brings the rich theory and a variety of powerful inference techniques of probabilistic graphical models to one’s disposal. This is what BAYESIL achieves by dividing the spectrum into interdependent blocks. Fig 4 shows a portion of the factor-graph for a simple defined mixture of 15 compounds. A factor graph is a graphical model with two types of nodes: 1) factors (corresponding to regions), and 2) variables (here, concentrations and chemical shifts). Each factor has arcs that point only to its associated variables.

**Fig 5:**
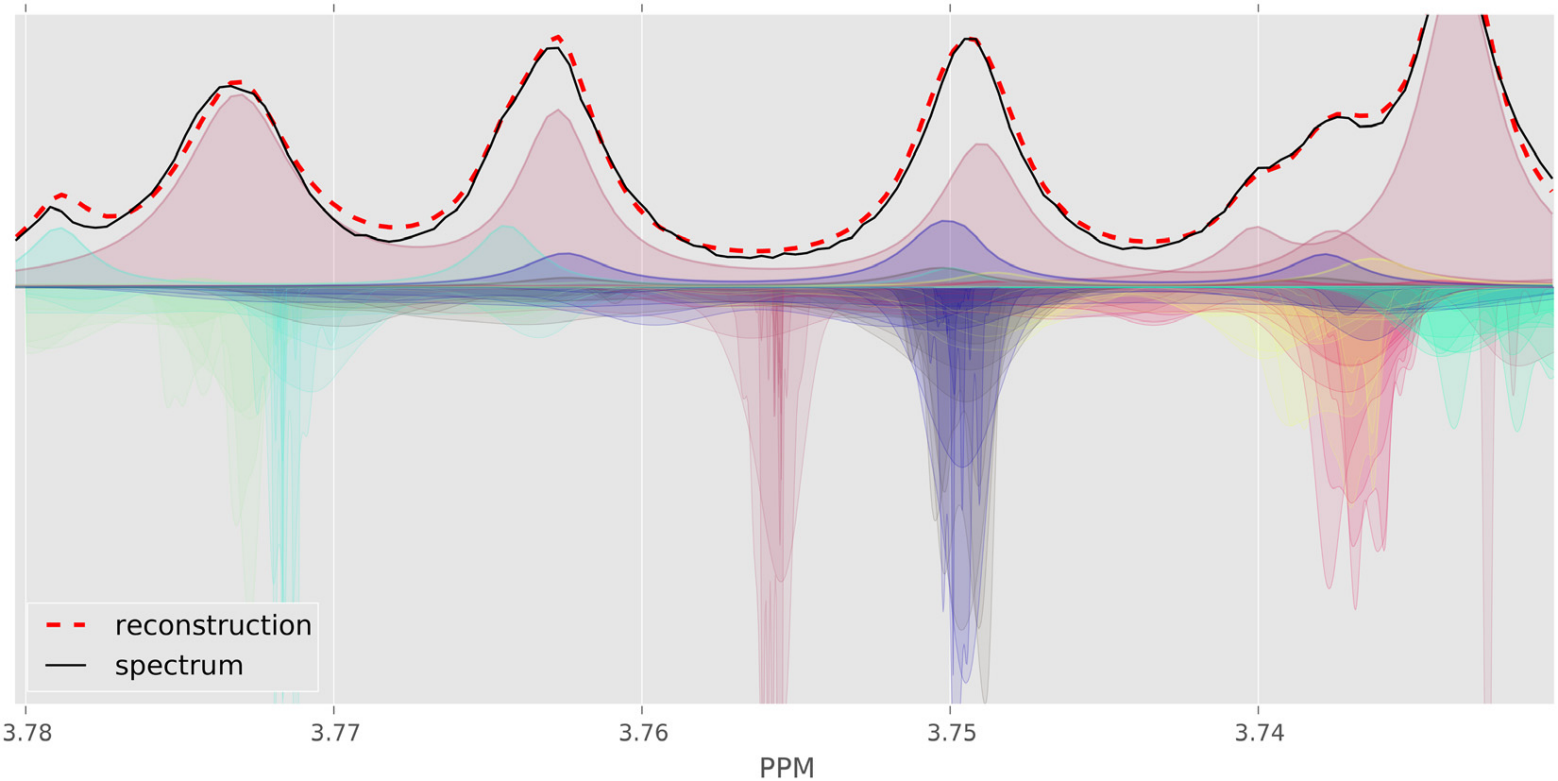
Evolution of Bayesil’s distributions for a small region of human serum spectrum. The plots above horizontal axis show the original spectrum (solid black), individual clusters as well as overall fit (dashed red). The curves below horizontal axis show the Bayesil’s distribution over chemical shift variables for each cluster (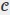), over 6 iterations of spectral deconvolution. The distributions become more peaked towards the correct center in each iteration. Distributions below the horizon have the color of their associated cluster.

BAYESIL uses a sequential Monte Carlo inference method [35] tailored to its inference problem. It defines a distribution over each concentration *ρ*_ℳ_ and shift variable *δ*_𝓒_. These distributions are gradually narrowed in each iteration until convergence, at which point the mode of the distributions approximates the most probable assignment. Here, the assignment to concentration variables ***ρ*** approximates the most probable metabolic profile. Fig 5 shows the evolution of distributions over the chemical shift variables over 6 iterations of spectral profiling. S2 Appendix gives details on BAYESIL’s spectral profiling procedure.

### Quantification

The concentrations that we obtain after spectral profiling are relative. BAYESIL uses a reference compound (*e.g.*, 4,4-dimethyl-4-silapentane-1-sulfonic acid, *a.k.a*. DSS or trisodium phosphate *a.k.a*. TSP) with known concentration, to obtain the absolute quantities. BAYESIL then estimates the “detection threshold” based on the signal to noise ratio (SNR) in each spectrum—*i.e.*, when the spectrum is noisy, this threshold is increase to provide a more confident identification and quantification. The SNR and therefore the detection threshold is directly related to the number of scans during spectral acquisition. For example, our biological serum samples in our experiments are produced using 128 scans and therefore most detection thresholds are ∼ 10*μ*M, while CSF samples that use 1024 scans often have threshold of less than 2*μ*M. However this threshold is not uniform across metabolites. BAYESIL also uses a relative factor in compound detectability; as some compound such as Choline are easy to identify and quantify at low concentrations while for some other compounds such as L-Asparagine, experts use a higher detection threshold.

### BAYESIL’s spectral library

We collected 1D ^1^ H NMR reference spectra for each of the compounds in BAYESIL’s spectral library using pure compounds obtained from the Human Metabolome Library [36], using a standard protocol (see the following subsection). The spectral library contains relevant information about each compound (ℳ) including individual peak clusters (𝓒) and peak amplitude positions and widths, as well as allowable chemical shift window ${\underline{\delta }}_{𝓒}\leq \delta_{𝓒}\leq {\bar{\delta }}_{𝓒}$ for each cluster 𝓒.

To analyze each biofluid, BAYESIL uses a specific spectral sub-library—here, one for serum and another one for CSF. The serum library consists of 50 NMR-detectable compounds from the human serum metabolome [37] while the CSF library consists of the 48 NMR-detectable compounds from the human CSF metabolome [38]. BAYESIL’s biofluid-specific databases include essentially all NMR-detectable metabolites (with concentrations > 5 *μ*M) in serum and CSF in healthy humans—*i.e.*, for normal human beings, without genetic inborn errors of metabolism (< 0.2% of the population) or exposures to lethal or near-lethal doses of drugs/poisons; see S3 Appendix. The use of biofluid-specific or organism-specific spectral libraries significantly improves the performance of the spectral fitting process as it reduces the number of possible explanations for each peak.

#### Sample preparation protocol

To produce each of the reference spectra for BAYESIL’s library, we first prepared stock solutions (1 mM to 100 mM) for each compound in 1 L in volumetric flasks. The metabolites were dissolved in 20 mM NaHPO_4_ (pH 7.0). These stock solutions were further diluted if necessary to obtain a final stock solution concentration of 1 mM. The final sample for NMR was prepared by transferring 1140 *μ*L to a 1.5 mL Eppendorf tube followed by the addition of 140 *μ*L D_2_O and 120 *μ*L of the reference standard solution (11.67 mM DSS (disodium-2,2-dimethyl-2-silapentane-5-sulphonate), 20 mM NaHPO_4_, pH 7.0). After confirming that the pH of the sample was between 6.8 and 7.2 (adjusting the buffer if necessary), we transferred 700 *μ*L to a standard NMR tube for spectral acquisition. All library ^1^ H NMR spectra were collected on both 500 MHz and 600 MHz Inova spectrometers equipped with 5 mm Z-gradient PFG probes. A standard presaturation ^1^ H-NOESY experiment(tnnoesy.c) was acquired at 25°C using the first increment of the presaturation pulse sequence. A 4 s acquisition time, a 100 ms mixing time, a 10 ms recycle delay and a 990 ms saturation delay were chosen. Thirty-two transients were acquired for samples collected at 600 MHz while 128 transients were acquired for all samples collected at 500 MHz. Eight steady state scans were employed and the presaturation pulse power was calibrated to provide a field width no greater than 80 Hz. Both the transmitter offset and the saturation pulse were centred on the water resonance and no suppression gradients were used. After spectral collection, the spectra were checked for quality and then analyzed using a locally developed spectral analysis tool to convert the spectra into a series of XML files. In producing the XML library, most peak clusters were given a default shift-window of 0.025 PPM, with the exception of few compounds such as histidine or citrate that are known to be highly pH-sensitive. For these we used a larger shift window as suggested by the experts. Both the synthetic and real biological spectral data were collected in the manner described above except for biological CSF in which 1024 scans were collected to compensate for dilution. For sample preparation, CSF was used as is, while serum was obtained after the blood had clotted for 30 min at 25°C and then passed through pre-rinsed 3000 MWCO Amicon Ultra-0.5 filters to remove remaining proteins. In each case 285 *μ*L of filtrate was obtained and 35 *μ*L of D_2_O and 30 *μ*L of buffer was added. A total of 350 *μ*L was then transferred to a suitable Sigma tube for NMR data acquisition. In the case of biological CSF, where less than 285 *μ*L was obtainable, the samples were diluted with sufficient H_2_O.

## Assessment

BAYESIL was assessed using 3 different types of spectral data sets over two different types of biofluids:

***(a)** Computer generated mixtures derived from its spectral library:* We generated 5 random serum and 5 random CSF spectra by sampling from the distribution of the measured concentration ranges of various compounds, and the probability of observing them in the mixture [37, 38]. The chemical shifts were also randomly sampled according to the chemical shift ranges from the corresponding spectral libraries. These correspond to “perfect” spectra, and are intended to assess the performance limits of BAYESIL under optimal conditions.

***(b)** Defined mixtures prepared in the laboratory:* We created 15 defined mixtures (5 defined mixture of serum, 5 defined mixture of CSF, 5 random mixture of compounds in both serum and CSF, involving > 60 compounds), using carefully measured pure compounds and freshly prepared solutions. These provide real spectral data that probably include common spectral and solution artifacts (baseline and phasing issues, minor spontaneous reaction products, contaminants, matrix or pH effects). This set was used to assess BAYESIL’s performance under well-controlled conditions.

***(c)** Biological serum and* CSF *samples:* We took human CSF and serum samples from previously studied samples that had been analyzed and quantified by NMR experts – here, 50 human serum and 5 human CSF samples. The set of compound mixtures was used to assess BAYESIL’s performance under realistic conditions with common spectral and solution artifacts. Although human CSF contains a smaller number of NMR-detectable compounds than human serum, it is more difficult to profile due to the lower concentration of metabolites. While both the biological samples and defined mixtures were thoroughly analyzed, their exact compound concentrations cannot be perfectly known.

Overall, we believe these 3 test sets provide a robust assessment of BAYESIL’s performance (as well as its limitations) under a wide range of conditions.

Given a spectrum of a mixture of compounds (with “true” concentrations {*ρ*_ℳ_}), BAYESIL returns its *estimates* of these concentrations $\left\{{\hat{\rho }}_{ℳ}\right\}$, which might be 0 if that compound is absent. We say a compound is a true positive if both ${\hat{\rho }}_{ℳ}$ and *ρ*_ℳ_ are positive—that is, greater than the detection threshold, and a true negative if both ${\hat{\rho }}_{ℳ}$ and *ρ*_ℳ_ are less than the threshold; in either case, BAYESIL’s prediction is considered correct. BAYESIL’s identification accuracy for a given spectrum is the ratio of correct labels (true positives plus true negatives) to the library size. BAYESIL’s “quantitative accuracy” describes how often its estimates ${\hat{\rho }}_{ℳ}$ were “close enough” to the true values *ρ*_ℳ_; note that simply computing $|{\hat{\rho }}_{ℳ}-\rho_{ℳ}|$ is not enough as this measure would basically only consider the compounds with high concentrations. We instead use the ${\text{median}}_{ℳ}\left(\frac{|\rho_{ℳ}-{\hat{\rho }}_{ℳ}|}{\max({\hat{\rho }}_{ℳ},\rho_{ℳ})}\right)$ as a measure of the percentage error in concentrations.

**Fig 6:**
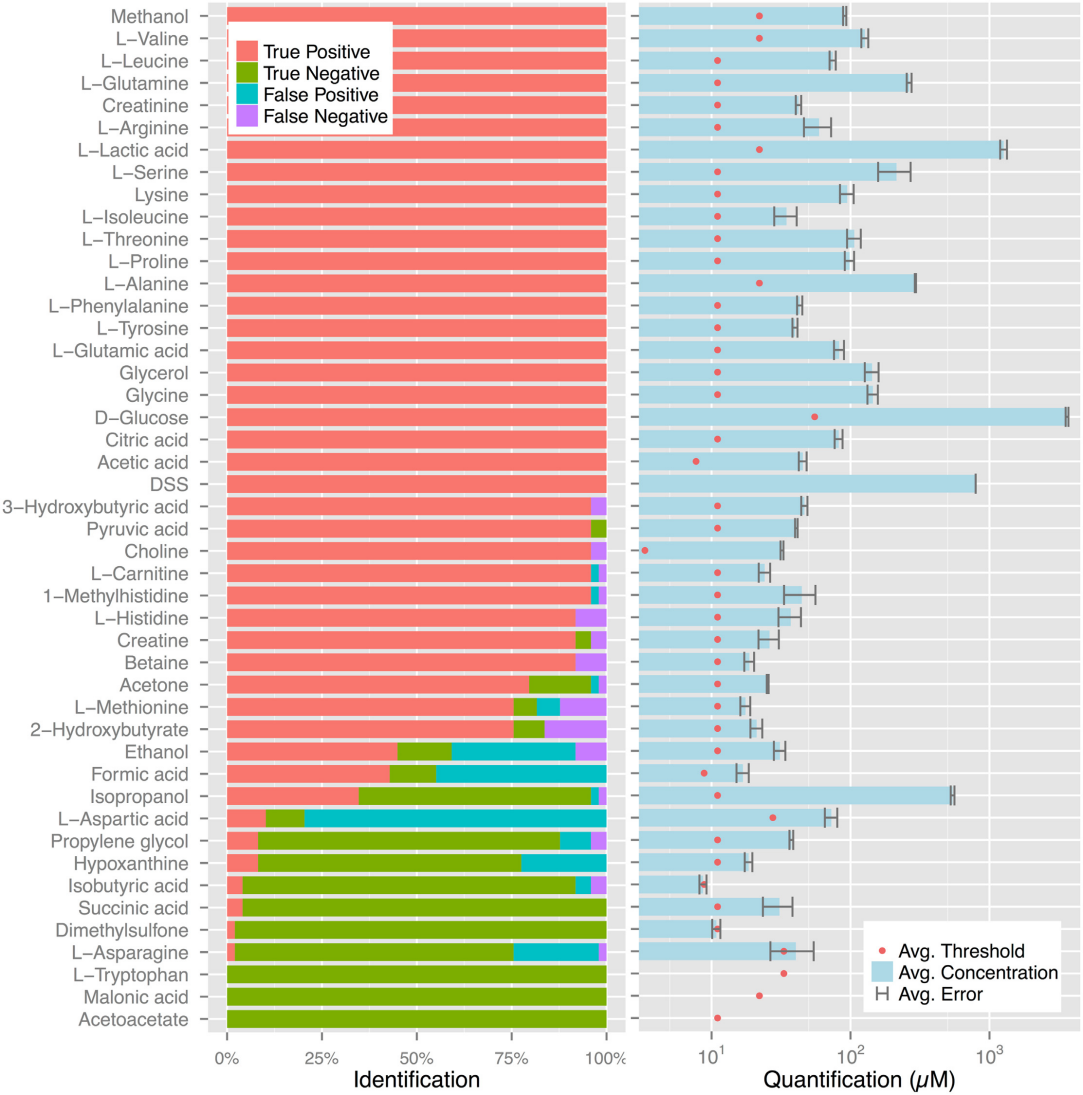
Bayesil’s quantification and identification. (left) Bayesil’s identification of individual compounds in 50 biological serum samples. (right) The average concentration for correctly identified compounds in the same samples. The error bars show the average difference between Bayesil and expert values for each compound and the red dots show the average detection threshold for the same compound.

Table 1 reports BAYESIL’s identification and quantification accuracies, for each of the tasks listed above; see Methods for exact definition of these accuracy measures. For the biological and lab synthesized samples, we assume the human expert’s assessment is correct, while for the computer generated spectra, the exact ground truth is known. Fig 6(left) reports the frequency of false/true positives/negatives for individual compounds in 50 serum samples. Fig 6(right) shows the average of *ρ*_ℳ_ for correctly identified compounds in 50 serum samples, as reported by BAYESIL, the average detection threshold for different compounds as well as the average difference ${\hat{\rho }}_{ℳ}-\rho_{ℳ}$, between BAYESIL and expert’s estimate for each compound.

**Table 1:** Identification and quantification accuracy of Bayesil and human expert on various data-sets.

		serum			CSF			complex
		biological	def. mix.	comp. gen.	biological	def. mix.	comp. gen.	def. mix.
BAYESIL	id. accuracy	.93 ± .04	.94 ± .02	.98 ± .01	.90 ± .04	.89 ± .03	.95 ± .03	.90 ± .02
	quant. accuracy	.89 ± .02	.90 ± .02	.98 ± .01	.91 ± .01	.90 ± .02	.94 ± .02	.88 ± .02
expert	id. accuracy	-	-	.91 ± .02	-	-	.87 ± .05	-
	quant. accuracy	-	-	.95 ± .01	-	-	.91 ± .04	-

These results on a diverse set of test data suggest that BAYESIL is often within 10% of the expert’s estimate, and where the ground truth is known, BAYESIL’s metabolic profile is often more accurate than the expert’s. BAYESIL’s web-page (http://www.bayesil.ca) provides a complete description of all of the studies reported above, showing the fits and the metabolic profiles obtained.

## Discussion

NMR is a particularly appealing platform for conducting metabolomics studies on biofluids as it is a rapid, robust, reproducible, non-destructive, and fully quantitative technique that requires minimial sample preparation. The main barrier delaying more prevalent use of metabolomics via NMR is the requirement for manual spectral profiling.

BAYESIL addresses this critical problem by providing fully automated spectral processing and deconvolution. Key to the high level of performance of BAYESIL is the use of biofluid-specific spectral libraries in its spectral fitting routines (*a.k.a*. targeted profiling). This need for prior knowledge about the typical composition of biofluid mixtures has motivated us, and others, to spend considerable efforts to determine the NMR-detectable metabolomes for many biofluids, including human plasma/serum [37], cerebrospinal fluid [38], human urine [39], saliva [40], milk [41] and rumen [42], mammalian cell extracts [43], bacterial cell extracts [44], cancer cells [45, 46], various juices [47] and and many other fluids or extracts. BAYESIL’s library is being actively expanded to allow its application to a more diverse set of biofluids.

Moreover BAYESIL is accurate and fast; on a commodity computer (*i.e.*, with a single 2.8 GHz CPU processor), BAYESIL typically takes less than 5 minutes to profile a serum or CSF spectrum with 90% accuracy. Over a sustained 24 hour period, BAYESIL should be able to process more than 200 spectra (vs. ∼ 20 spectra/day for a human expert) and accurately identify-&-quantify approximately 50 compounds per spectrum. This makes BAYESIL the first system to enable high-throughput metabolomics, since a single CPU is able to output more than 5000 metabolite measurements a day. In comparison, the state-of-the-art semi-automated software takes hours or days to achieve much less accuracy on the same samples (see S1 Appendix).

BAYESIL has its own limitations; for instance its accuracy has so far been only validated for serum and CSF. Furthermore, it only works if these biofluids have been prepared and collected as prescribed in this paper. Likewise, if BAYESIL were to be used on certain biofluids such as cell extracts that contain chemically similar compounds (*i.e.*, Adenine, Adenosine, AMP, ADP, *etc.*) the lack of chemical shift uniqueness could confuse the system. Additionally, compounds with overlapping single resonances (*e.g.*, Acetate, Acetone, Succinate, Pyruvate *etc.*) can potentially be misidentified and/or misquantified. However, these situations do not occur in serum and CSF.

Overall, we believe that removing the automation barrier will have a significant, positive impact on NMR spectroscopy and NMR-based metabolomics. In particular, this system will enable medical researchers and clinicians to quickly and accurately obtain metabolic profiles of patient biofluids, which will ultimately lead to better diagnoses and treatments. BAYESIL is freely available for users to perform metabolic profiling of 1D ^1^ H NMR spectra of serum, plasma and CSF.

## Supporting Information

S1 Appendix Other NMR-analysis software tools. This appendix reviews the existing software packages for NMR analysis, their capabilities and limitations. Here we also compare BAYESIL against batman, a widely used software package for semi-automated targeted profiling.
(PDF)

S2 Appendix Details of BAYESIL’s spectral profiling. This appendix elaborates construction of the factor graph and BAYESIL’s inference procedure for spectral profiling.
(PDF)

S3 Appendix List of NMR-detectable compounds in serum and CSF. (PDF)

S1 Dataset This appendix contains raw spectra studied in this paper and their metabolic profiles as reported by the expert and Bayesil. (ZIP)
