# Correction: Accurate, Fully-Automated NMR Spectral Profiling for Metabolomics

**DOI:** 10.1371/journal.pone.0132873

**Published:** 2015-07-29

**Authors:** Siamak Ravanbakhsh, Philip Liu, Trent C. Bjorndahl, Rupasri Mandal, Jason R. Grant, Michael Wilson, Roman Eisner, Igor Sinelnikov, Xiaoyu Hu, Claudio Luchinat, Russell Greiner, David S. Wishart

The third author’s name is spelled incorrectly. The correct name is: Trent C. Bjorndahl. The correct citation is: Ravanbakhsh S, Liu P, Bjorndahl TC, Mandal R, Grant JR, Wilson M, et al. (2015) Accurate, Fully-Automated NMR Spectral Profiling for Metabolomics. PLoS ONE 10(5): e0124219. doi:10.1371/journal.pone.0124219
