# Supplementary material for: Accurate, Fully-Automated NMR Spectral Profiling for Metabolomics
Source: PLoS One. 2015 May 27;10(5):e0124219. doi: 10.1371/journal.pone.0124219 (PMC4446368; doi:10.1371/journal.pone.0124219)
Supplement: S1 Appendix — This appendix reviews the existing software packages for NMR analysis, their capabilities and limitations. Here we also compare BAYESIL against batman, a widely used software package for semi-automated targeted profiling. (PDF) [file pone.0124219.s001.pdf]

Other NMR-analysis software tools

Several software packages have been developed to facilitate NMR spectral processing, compound identification and quantification. While some facilitate two dimensional NMR spectral processing and compound identification (*e.g.*, Colmar [1],Metabominer [2], dataChord (One Moon Scientific)), none are completely automated and provide compound quantification. Furthermore, because modern metabolomics NMR studies require the analysis of a tremendous amount of spectral data, the time required to collect 2D NMR spectra makes their implementation prohibitive not only due to their inherent inability to facilitate high-throughput studies but also due to complicating factors such as sample degradation within the spectral acquisition time frame. Thus, more effort has been directed at developing software tools for 1D NMR.

Some tools such as NMRGlue [3] and NMRPipe [4] provide basic processing functionalities. (In fact, BAYESIL uses NMRGlue [3] for handling different input/output data formats.)

We focus on software package capable of handling high-throughput 1D metabolomics NMR data. An ideal tool here should have the following features: (1) *fully automated* – in both spectral processing and compound identification and quantification; (2) *flexible and customizable* – capable of analyzing a wide (and extendable) range of different biological fluids; (3) *ubiquitous* – can accommodate input from different NMR vendors, multiple spectrometer frequencies; and of course (4) *accurate.*

Various software packages have made incremental steps toward achieving these goals. Of the 19 that we could identify, only a handful provide some degree of automated identification and/or quantification (1): Colmar [1], BQuant [5], HiRes [6], Autofit [7], CEED [8], BATMAN [10], QMTLS [12], Juice Screener, Wine Screener and Metabolic Profiler (Bruker Corporation) and Chenomx NMR Suite (Chenomx Inc.). This task (of spectral profiling and metabolite profiling) has been tackled with a variety of algorithmic approaches – including simple text file matching, binning [5], principal component analysis and non-negative matrix factorization [1,6], combinations of simulated annealing and gradient descend ([7], Chenomx NMR Suite (Chenomx Inc.)), cross entropy method [8] and Monte Carlo techniques [10]. However only a few of these software packages provide automated spectral processing (LC-model [11],Juice Screener and Wine Screener).

In terms of flexibility and customizability (2), some software packages do utilize large data-sets (HMDB [14], BMRB [15] or MMCD [16]) but they still require the user to select a subset of the compounds, and/or do not provide quantification [1,2,9]. Others are specialized to particular mixtures [11], Wine Screener, Juice Screener and Vantera (LipoScience Inc.)) and none can accurately quantify complex mixtures (with > 50 compounds). Moreover, many of these software packages are specific to a particular instrument (*e.g.*, BATMAN, CEED, QMTLS, Crockford, Wine Screener, Juice Screener, dataChord and Vantera).

It is often difficult to access accuracy (4), as the descriptions of many software tools do not provide any assessment (*e.g.*, Juice Screener, Wine Screener and Metabolic Profiler (Bruker Corporation), Colmar, LC-model) and many systems have been assessed merely on very simple mixtures (*e.g.*, Metabominer [2], Metabohunder [9], BATMAN [10],lipoprofiler/Vantera (LipoScience Inc.)) or simple spike-in experiments [5,12,13].

Comparison with BATMAN

To date, the largest number of metabolites that has been automatically identified and quantified using publicly available software is 26 compounds, by BATMAN [10]. However, an analysis of this magnitude required several hours of CPU time to process a single spectrum. Furthermore, BATMAN also requires a human expert to perform many of the preliminary steps. We compared BAYESIL to BATMAN on simple computer-generated mixtures, involving 5, 10 and 20 compounds selected from BATMAN's library, as well as a preprocessed human serum sample. For the computer generated spectra, both BATMAN and BAYESIL used identical libraries containing only the relevant compounds. BATMAN achieved $85 - 87\%$ quantification

accuracy for the computer-generated mixtures but took 2-9 hours to run, while in all cases BAYESIL achieved $> 98\%$ accuracy in less than 3 minutes. For the serum spectra, BAYESIL used a library of 50 compounds, while BATMAN used a subset of 40 compounds that its library has in common with the serum metabolome. BATMAN took 19 hours to analyze the serum spectrum and identified all the compounds in its library (resulting in 85% identification accuracy) and only 8% quantification accuracy compared to BAYESIL which took 5 minutes to achieve 98% identification and 90% quantification accuracy.

To summarize, BAYESIL is the only 1D ^{1}H NMR interpretation system that is completely automated (both preprocessing and deconvolution) for a wide range of complex mixtures (*i.e.*, all mammalian biofluids covered by its current library; see *www.bayesil.ca*), involving > 60 compounds. Moreover, it is efficient, general, accurate and publicly available.

References

1. Robinette, S. L., Zhang, F., Bruschweiler-Li, L., Bruschweiler, R. (2008). Web server based complex mixture analysis by NMR. *Anal Chem, 80(10), 3606-3611.*

2. *Xia, J., Bjorndahl, T. C., Tang, P., Wishart, D. (2008). MetaboMinersemi-automated identification of metabolites from 2D* NMR *spectra of complex biofluids. BMC bioinformatics, 9(1), 507.*

3. *Helmus, J. J., Jaroniec, C. P. (2013) Nmrglue: an open source Python package for the analysis of multidimensional NMR data. Journal of biomolecular NMR, 55(4), 355-367.*

4. *Delaglio, F. et al. (1995). NMRPipe: a multidimensional spectral processing system based on UNIX pipes. J Biomol NMR, 6(3), 277-293.*

5. *Zheng, C., Zhang, S., Ragg, S., Raftery, D., Vitek, O. (2011). Identification and quantification of metabolites in 1H NMR spectra by Bayesian model selection. Bioinformatics, 27(12), 1637-1644.*

6. *Zhao, Q., Stoyanova, R., Du, S., Sajda, P., Brown, T. R. (2006). HiResa tool for comprehensive assessment and interpretation of metabolomic data. Bioinformatics, 22(20), 2562-2564.*

7. *Mercier, P., Lewis, M. J., Chang, D., Baker, D., Wishart, D. (2011). Towards automatic metabolomic profiling of high-resolution one-dimensional proton NMR spectra. J Biomol NMR, 49(3-4), 307-323*

8. *Ravanbakhsh, S., Poczos, B., Greiner, R. (2010). A Cross-Entropy method that optimizes partially decomposable problems: a new way to interpret NMR spectra. Proc Conf AAAI Artif Intell*

9. *Tulpan, D., Leger, S., Belliveau, L., Culf, A., Cuperlovic-Culf, M. (2011). MetaboHunter: an automatic approach for identification of metabolites from 1H-NMR spectra of complex mixtures. BMC bioinformatics, 12(1), 400.*

10. *Hao, J., Astle, W., De Iorio, M., Ebbels, T. M. (2012). BATMANan R package for the automated quantification of metabolites from nuclear magnetic resonance spectra using a Bayesian model. Bioinformatics, 28(15), 2088-2090.*

11. *Provencher, S. W. (1993). Estimation of metabolite concentrations from localized in vivo proton NMR spectra.Magnetic Resonance in Medicine, 30(6), 672-679.*

12. *Mihaleva, V. V. et al. (2014). Automated quantum mechanical total line shape fitting model for quantitative NMR-based profiling of human serum metabolites. Anal Bioanal Chem, 406(13), 3091-3102.*

13. *Crockford, D. J., Keun, H. C., Smith, L. M., Holmes, E., Nicholson, J. K. (2005). Curve-fitting method for direct quantitation of compounds in complex biological mixtures using 1H NMR: application in metabonomic toxicology studies. Anal Chem, 77(14), 4556-4562.*

14. *Wishart et al. (2007). HMDB: the human metabolome database. Nucleic Acids Res, 35(suppl 1), D521-D526.*

15. *Eldon L. Ulrich et al. (2008) BioMagResBank, Nucleic Acids Res, 36, D402-D408*

16. *Q. Cui et al. (2008) Metabolite identification via the Madison Metabolomics Consortium Database", Nat Biotechnol, 26,162*
