# Supplementary material for: Accurate, Fully-Automated NMR Spectral Profiling for Metabolomics
Source: PLoS One. 2015 May 27;10(5):e0124219. doi: 10.1371/journal.pone.0124219 (PMC4446368; doi:10.1371/journal.pone.0124219)
Supplement: S2 Appendix — This appendix elaborates construction of the factor graph and BAYESIL’s inference procedure for spectral profiling. (PDF) [file pone.0124219.s002.pdf]

Details of BAYESIL's spectral profiling

Here, we review and re-express BAYESIL's objective and optimization procedure in more details. Recall that an NMR spectrum for a compound $\mathcal{M}$ is a collection of one or more *Lorentzian* peaks formed into one or more clusters – that is, each compound $\mathcal{M}$ is a set of clusters $\{\mathcal{C}_k\}$, where each cluster $\mathcal{C}_k$ is set of peaks, and each peak is defined by a triple, $\boldsymbol{\theta} = (\theta_1, \theta_2, \theta_3)$ corresponding to its height, center and width (at half height) respectively. The height at x due to this peak is defined by the Lorentzian function $\mathsf{q}(x; \boldsymbol{\theta}) = \frac{\theta_1 \theta_3}{\theta_3 + 4(\theta_2 - x)^2}$. Letting $\mathcal{X}$ refer to the entire spectrum (*e.g.*, from -1 to 13 PPM when referenced against the DSS peak), the height of the spectrum of a pure compound $\mathcal{M}$ at each location $x \in \mathcal{X}$, is the sum of all the peaks in clusters of $\mathcal{M}$

$$\widehat{\mathsf{s}}(x\,; \mathcal{M}, \rho_{\mathcal{M}}, \boldsymbol{\delta}_{\mathcal{M}}) \quad = \quad \rho_{\mathcal{M}} \sum_{\mathcal{C} \in \mathcal{M}} \sum_{\boldsymbol{\theta} \in \mathcal{C}} \mathsf{q}(x - \delta_{\mathcal{C}}; \boldsymbol{\theta}) \tag{1}$$

where $\rho_{\mathcal{M}}$ is the concentration of this compound and $\boldsymbol{\delta}_{\mathcal{M}} = \{\delta_{\mathcal{C}} \,|\, \mathcal{C} \in \mathcal{M}\}$ is the set of chemical shifts for the clusters associated with this compound.

An NMR spectrum is essentially a linear combination of the peaks in its component compounds: that is, the height at each PPM value x of a mixture spectrum is just the sum of the contributions of each compound. This means, given the concentrations of the compounds $\boldsymbol{\rho} = \{\rho_{\mathcal{M}}\}$, and the chemical shifts $\boldsymbol{\delta} = \bigcup_{\mathcal{M}} \boldsymbol{\delta}_{\mathcal{M}}$ of the clusters associated with these compounds, we can then "draw" an NMR spectrum – *i.e.*, $\widehat{\mathsf{s}}(x\,; \boldsymbol{\rho}, \boldsymbol{\delta}) \;=\; \sum_{\mathcal{M}} \widehat{\mathsf{s}}(x\,; \mathcal{M}, \rho_{\mathcal{M}}, \boldsymbol{\delta}_{\mathcal{M}})$.

The spectral profiling challenge, in general, is the reverse process: Given a set of compounds $\{\mathcal{M}_1, \ldots, \mathcal{M}_r\}$ with associated signatures (*i.e.*, $\boldsymbol{\theta}$ values of their peaks, organized in clusters) and the observed spectrum $\mathsf{s}(\cdot)$, find the "best" combination of concentrations $\boldsymbol{\rho}$ and shifts $\boldsymbol{\delta}$ to fit that spectrum.

Loss function

To determine which values are best, for now, BAYESIL uses a complex loss function. This loss function is the (square of the) difference of the heights between the observed spectrum $\mathsf{s}(\cdot)$ and the reconstructed spectrum $\widehat{\mathsf{s}}(\cdot\,; \boldsymbol{\delta}, \boldsymbol{\rho})$ plus a additional penalties for the derivatives of the difference:

$$\ell_{\mathcal{X}}\left(\mathsf{s}(\cdot), \widehat{\mathsf{s}}(\cdot\,; \boldsymbol{\rho}, \boldsymbol{\delta})\right) = \sum_{c \in \{0,1,2,3\}} \gamma_c \int_{\mathcal{X}} \left(\frac{\partial^c}{\partial x^c} (\mathsf{s}(x) - \widehat{\mathsf{s}}(x\,; \boldsymbol{\rho}, \boldsymbol{\delta})) \right)^2 \mathrm{d}x \tag{2}$$

where the subscript $\mathcal{X}$ indicates that this loss function applies to the entire spectrum. Here, the integral for $c = 0$ corresponds to sum of squared errors and $c \geq 1$ enforce the smoothness of the difference between $\mathsf{s}(\cdot)$ and $\widehat{\mathsf{s}}(\cdot)$ (*a.k.a.* total variation norm). The scalars γ_c weight the relative importance of these terms.

Our task is to find the values of

$$[\boldsymbol{\rho}^*,\ \boldsymbol{\delta}^*] \quad = \quad \arg_{\boldsymbol{\rho}, \boldsymbol{\delta}} \min \quad \ell_{\mathcal{X}}\left(\mathsf{s}(\cdot),\, \widehat{\mathsf{s}}(\cdot\,; \boldsymbol{\rho}, \boldsymbol{\delta})\right) \tag{3}$$

that minimize this loss function.

Construction of regions

The key innovation of BAYESIL is how it minimizes this highly non-linear loss function, efficiently. In particular, BAYESIL "factors" this large task into a set of inter-related smaller tasks. The construction of the spectral regions is based on the observation that the influence of each peak (and hence of each spectral cluster) is significant over only a relatively small region of the spectrum. To estimate this "region of effect" for each cluster, BAYESIL first obtains an upper-bound $\overline{\rho}_{\mathcal{M}} \geq \rho_{\mathcal{M}}$ on the concentration of each compound. This upper-bound is also used in performing approximate inference. For each compound $\mathcal{M}$, this is the minimum of the upper-bounds obtained using each of its clusters $\mathcal{C} \in \mathcal{M}$: $\overline{\rho}_{\mathcal{M}} = \min_{\mathcal{C} \in \mathcal{M}} \overline{\rho}_{\mathcal{C}}$.

The upper-bound from each cluster ($\overline{\rho}_{\mathcal{C}}$) is obtained by progressively shifting the signature for that cluster under the spectrum and finding the maximum value that it can take, assuming all the other compounds and clusters are absent:

$$\overline{\rho}_{\mathcal{C}} \quad = \quad \max_{\delta_{\mathcal{C}}} \min_{x} \frac{\mathsf{s}(x)}{\mathsf{s}_{\mathcal{C}}(x; 1, \delta_{\mathcal{C}})}$$

where $\mathsf{s}_{\mathcal{C}}(., 1, \delta_{\mathcal{C}})$ is the signature of cluster $\mathcal{C}$ as defined by the set of all its peaks (see eq(**1**)) assuming a unit concentration and allowing $\delta_{\mathcal{C}}$ to vary in a small window $[\underline{\delta}_{\mathcal{C}}, \overline{\delta}_{\mathcal{C}}]$ around the center defined by the library.

Now that we have an upper-bound on concentrations, using an example from figure 3(left) in the main manuscript we show why the region of effect for each cluster is bounded. In this figure, the center ($\delta_{L-Isolucine(1)}$) for the first cluster of *L-Isoleucine* can only appear in the interval [0.9130, 0.9380] PPM; as its concentration is at most $\overline{\rho}_{L-Isoleucine} = 95\mu$M, its contribution to any point outside [.8563, .9954] will be five times less than the estimated *noise-level* of this spectrum, where the noise-level is estimated as the standard deviation of the spectrum $\mathsf{s}(\cdot)$ over all of the baseline points. We can therefore identify this L-Isoleucine cluster with the interval [.8563, .9954]. Note this cluster includes 3 peaks. In general, the range of a cluster spans the set of peaks that it contains.

Now consider a function that maps each point in the spectrum to the set of clusters that might affect the height at this location. That is, given the set of compounds $\{\mathcal{M}\}$ that might appear in a particular biofluid (*e.g.*, the 48 that can appear in CSF), we can identify each PPM location $x \in \mathcal{X}$ with the small set of clusters that might influence it, $\mathcal{C}(x)$.

We can then partition the spectrum into disjoint contiguous regions, $\{\mathcal{X}_I\}$, where every PPM location in each $\mathcal{X}_I$ involves exactly the same subset of clusters – *i.e.*, for any pair of points $x_1, x_2 \in \mathcal{X}_I$, we know that $\mathcal{C}(x_1) = \mathcal{C}(x_2)$.

For example in figure 3(left) in the main manuscript, the region $\mathcal{X}_{[.8563,9289]}$ from 0.8563 to 0.9289 PPM might include significant contributions from the first cluster of 2-Hydroxybutyrate, the first cluster of L-Isoleucine and/or the first cluster of L-Leucine. The region immediately to its left (from 0.9289 to 0.9370 PPM) includes these and also a cluster of L-Valine, and the one to the right (from 0.8563 to 0.87526 PPM) does not include L-Isoleucine.

As the loss function $\ell(\cdot, \cdot)$ is additive over the domain $\mathcal{X}$, we can rewrite the optimization of eq(**3**) as the sum of the losses for each of the regions $\mathcal{X}_I$:

$$[\boldsymbol{\rho}^*,\ \boldsymbol{\delta}^*] \ = \ \arg_{\boldsymbol{\rho},\boldsymbol{\delta}} \min \sum_I \ell_{\mathcal{X}_I}\big(\,\mathsf{s}(\cdot),\ \widehat{\mathsf{s}}(\cdot\,; \boldsymbol{\rho}_I, \boldsymbol{\delta}_I)\,\big) \qquad (4)$$

Now recall that each region $\mathcal{X}_I$ involves relatively few compounds and clusters. This suggests a preliminary step of simply "solving" each region, by itself: *i.e.*, find the best centers for the clusters in that region $\boldsymbol{\delta}_I$, and the best concentrations for the associated compounds $\boldsymbol{\rho}_I$, which collectively minimize the loss over the PPM-interval $\mathcal{X}_I$. This simple approach is fast, as it involves relatively few variables and a limited range of PPM-values. Unfortunately, this does not produce the overall correct answer – that is, each region has an opinion about the concentration and shift values of its cluster, and when two (or more) regions each involve the same variable, they must both agree on its value.

To address this problem, we take a probabilistic approach, viewing the task of minimizing the loss function eq(**2**) as finding the "Maximum a Posteriori" (MAP) assignment – *i.e.*, the assignment to all of the cluster-shift and compound-concentration $[\boldsymbol{\delta}, \boldsymbol{\rho}]$ variables that makes the observed data as likely as possible. Here, the Boltzmann formula gives the probabilistic interpretation of the loss (*a.k.a.* the energy)

$$\mathbb{P}(\,\boldsymbol{\rho}, \boldsymbol{\delta}\,|\,\mathsf{s}(\cdot)\,) \ = \ \frac{1}{Z}\exp\left\{-\frac{1}{T}\ell_{\mathcal{X}}\big(\,\mathsf{s}(\cdot),\ \widehat{\mathsf{s}}(\cdot\,; \boldsymbol{\rho}, \boldsymbol{\delta})\,\big)\right\} \qquad (5)$$

where Z is the normalization constant and T is known as the "temperature" parameter,

Using the decomposition of loss over regions (eq(**4**)) we can write this distribution in factored form

$$\mathbb{P}(\,\boldsymbol{\rho}, \boldsymbol{\delta} \,|\, \mathsf{s}(\cdot)\,) = \frac{1}{Z} \prod_I \mathsf{f}_I(\boldsymbol{\rho}_I, \boldsymbol{\delta}_I)$$

$$\mathsf{f}_I(\boldsymbol{\rho}_I, \boldsymbol{\delta}_I) = \exp\left\{ \; - \frac{1}{T} \ell_{\mathcal{X}_I}\big(\, \mathsf{s}(\cdot),\; \widehat{\mathsf{s}}(.; \boldsymbol{\rho}_I, \boldsymbol{\delta}_I)\,\big) \right\} \tag{6}$$

As we explained in the main manuscript, this decomposition of the distribution $\mathbb{P}(\,\boldsymbol{\rho}, \boldsymbol{\delta} \,|\, \mathsf{s}(\cdot)\,)$ can be represented using a probabilistic graphical model, known as a factor graph [4,5] which is a graph with two types of nodes: 1) factors (corresponding to regions or f_I), and 2) variables (here, concentrations and chemical shifts). Each factor has arcs that point only to its associated variables. BAYESIL performs approximate inference over this factor-graph to find the most probable assignment.

Sequential importance sampling in BAYESIL

Recall that $\boldsymbol{\rho}_I$ and $\boldsymbol{\delta}_I$ denote the set of shift and concentration values for all the clusters and associated compounds that can appear in the region $\mathcal{X}_I$; we let $\boldsymbol{\mu}_I = [\boldsymbol{\rho}_I, \boldsymbol{\delta}_I]$ denote the set of variables of both both types. Since the loss function $\ell(\cdot, \cdot)$ is additive over domain the $\mathcal{X}$, we can rewrite the optimization of eq(**3**) in exponential form as:

$$\boldsymbol{\mu}^* \;=\; \arg_\mu \max \prod_I \mathsf{f}_I(\boldsymbol{\mu}_I)$$

$$\mathsf{f}_I(\boldsymbol{\mu}_I) = \exp\left\{ \; - \frac{1}{T} \ell_{\mathcal{X}_I}(\, \mathsf{s}(\cdot),\, \widehat{\mathsf{s}}(.; \boldsymbol{\mu}_I)\,) \right\} \tag{7}$$

where each factor f_I is basically the exponential of the negative loss function ($-\ell_{\mathcal{X}_I}$) over the region $\mathcal{X}_I$ and T is the temperature. Here a factor node f_I is connected to all of its associated variables $\mu_i \in \mu_I$ in the factor-graph. In the following, we use $\partial \mu_i = \{\mathsf{f}_I \mid \mu_i \in \boldsymbol{\mu}_I\}$ to refer to all the factors that are adjacent to variable μ_i in the factor-graph.

Inference in this factor-graph is challenging as its factors can each depend on a large number of continuous variables. This means that the most basic task of (conditional) *sampling* from a factor is unfeasible and we cannot use Glauber dynamics [2]. This is further complicated by multi-modality of the factors, which prevents the use of parametric densities and inference techniques such as Gaussian Belief Propagation [5] or (primal and dual) decomposition methods that require convex sub-problems [1]. BAYESIL uses a non-parametric sequential Monte Carlo method that is closely related to sequential importance sampling and particle filters. The following is a step-by-step explanation of this inference procedure.

BAYESIL models a distribution $\mathbb{P}(\mu_i)$ over individual variables, non-parametrically: as a set of "particles". These particles, $\mu_i[n]$ for $1 \leq n \leq N$, collectively serve to approximate the target distribution. In all the experiments described in the manuscript we use $N = 10,000$ such particles, however using a larger number can increase accuracy at the cost of increased run-time.

For each n, the joint set of particles for all variables, $\boldsymbol{\mu}[n] = [\boldsymbol{\rho}[n], \boldsymbol{\delta}[n]]$, corresponds to a complete spectrum – *i.e.*, $\widehat{\mathsf{s}}(x\,; \boldsymbol{\rho}[n], \boldsymbol{\delta}[n]) \;=\; \sum_{\mathcal{M}} \widehat{\mathsf{s}}(x\,; \mathcal{M}, \rho_{\mathcal{M}}[n], \boldsymbol{\delta}_{\mathcal{M}}[n])$ – which means we can compute its loss (eq(**2**)). BAYESIL calculates the loss for each region $\mathcal{X}_I$, $\mathsf{f}_I(\mu_I[n])$ (eq(**7**)). Since each variable μ_i appears in many regions $\partial\mu_i$, we can "credit" each assignment $\mu_i[n]$ with the loss over all such regions. This allows us to compute a "weight" for each variable μ_i and for each particle n. BAYESIL then uses these weighted sets of particles to produce a new distribution for the variable μ_i, one that prefers values that have less loss. BAYESIL then iterates, using this new distribution, until convergence.

More specifically, BAYESIL first assigns each of the variables μ_i to an initial distribution of values $\mathbb{P}^{(0)}(\mu_i)$ – *e.g.*, $\delta_{L-Isoleucine(1)}$ is drawn uniformly from its chemical shift range [0.9130, 0.9380] PPM,

and $\rho_{L-Isoleucine(1)}$ is drawn uniformly from its range [0, 95] μM (see Appendix III for the procedure to estimate the upper bound $\overline{\rho}_{L-Isoleucine(1)} = 95\mu$M). It then iterates $t = 0, 1, 2, ...$ over the following four steps:

Step1: It draws N =10,000 particles from each $\mathbb{P}^{(t)}(\mu_i)$ independently, producing 10,000 complete assignment to these variables $\boldsymbol{\mu}^{(t)}[n] = [\boldsymbol{\rho}^{(t)}[n], \boldsymbol{\delta}^{(t)}[n]]$ (for $n = 1, 2, ...$,10,000), from its current product distribution.

Step2: For each joint particle $\boldsymbol{\mu}^{(t)}[n]$: For each factor I, BAYESIL computes the loss associated with this region, $\ell_I(\boldsymbol{\mu}_I[n]^{(t)})$, and then $\mathsf{f}_I(\boldsymbol{\mu}_I[n]^{(t)})$ using eq(**7**).

Step3: Recall each variable μ_i belongs to a set of regions (each corresponding to a factor), $\partial\mu_i$. BAYESIL then implicitly identifies $\mu_i[n]^{(t)}$ with the loss associated with all corresponding regions. This is achieved by defining a weight

$$\omega(\mu_i[n]^{(t)}) \propto \frac{\prod_{\mathsf{f}_I \in \partial\mu_i} \mathsf{f}_I(\boldsymbol{\mu}_I[n]^{(t)})}{\mathbb{P}^{(t)}(\mu_i[n]^{(t)})} \tag{8}$$

where $\mathbb{P}^{(t)}(\cdot)$ is the distribution used for sampling – *i.e.*, importance sampling weight.

Step4: Finally, BAYESIL produces a "new" distribution for each variable μ_i, using kernel density estimation (KDE) over a weighted set of the N particles $\boldsymbol{\mu}[n]^{(t)}$ $1 \leq n \leq N$ to represent the marginal distribution $\mathbb{P}^{(t+1)}(\cdot)$ over each variable $\mu_i \in \boldsymbol{\mu}$:

$$\mathbb{P}^{(t+1)}(\mu_i) \;\propto\; \sum_{n=1}^{N} \omega(\mu_i[n]^{(t)}) \; \mathsf{k}\left(\frac{\mu_i - \mu_i[n]^{(t)}}{h}\right)$$

where $\mathsf{k}(\cdot)$ is a kernel function (*e.g.*, a Gaussian) and the kernel bandwidth h is estimated from the data [3].

BAYESIL then checks for convergence; if convergence occurs, it returns the mode of individual distribution as its approximation to the MAP assignment. If not, it returns to Step1.

Note the temperature parameter T (used in eq(**7**) which appears in eq(**8**)) is gradually reduced from a large value towards zero per iteration. Figure 4 (in the main manuscript) is basically showing the evolution of KDEs over the chemical shift variables ($\mathbb{P}^{(t)}(\delta_{\mathcal{C}})$ for $t \in \{1, \ldots, 6\}$) over 6 iterations of spectral profiling.

In practice, BAYESIL ignores the importance sampling weights. This biases $\mathbb{P}^{(t+1)}(\mu_i)$ towards $\mathbb{P}^{(t)}(\mu_i)$, but significantly reduces the variance and the computation time. Since we are interested in the mode (rather than the marginals) of $\mathbb{P}(\mu_i)$, this trade-off is favourable, as the bias of the previous estimate is mostly towards more probable assignments.

References

1. Boyd, S. P., Vandenberghe, L. (2004). Convex optimization. *Cambridge university press.*

2. *Mezard, M., Montanari, A. (2009). Information, physics, and computation. Oxford University Press.*

3. *Silverman, B. W. (1986). Density estimation for statistics and data analysis (Vol. 26). CRC press.*

4. *Kschischang, F. R., Frey, B. J., Loeliger, H. A. (2001). Factor graphs and the sum-product algorithm. Information Theory, IEEE Transactions on, 47(2), 498-519.*

5. *Koller, D., Friedman, N. (2009). Probabilistic graphical models: principles and techniques. MIT press.*
