# Supplementary material for: Accurate, Fully-Automated NMR Spectral Profiling for Metabolomics
Source: PLoS One. 2015 May 27;10(5):e0124219. doi: 10.1371/journal.pone.0124219 (PMC4446368; doi:10.1371/journal.pone.0124219)
Supplement: S3 Appendix — (PDF) [file pone.0124219.s003.pdf]

List of NMR-detectable compounds in serum and CSF

Our serum library includes the following metabolites plus DSS and TSP:
1-Methylhistidine, 2-Hydroxybutyric acid, Acetic acid, Betaine, Acetoacetic acid, L-Carnitine, Creatine, Citric acid, Choline, Ethanol, D-Glucose, Glycine, Glycerol, Formic acid, L-Glutamic acid, Hypoxanthine, L-Tyrosine, L-Phenylalanine, L-Alanine, L-Proline, L-Threonine, L-Asparagine, L-Isoleucine, L-Histidine, L-Lysine, L-Serine, L-Lactic acid, L-Aspartic acid, Ornithine, Pyruvic acid, Succinic acid, Urea, 3-Hydroxybutyric acid, L-Arginine, Creatinine, L-Glutamine, L-Leucine, Malonic acid, L-Methionine, Isopropyl alcohol, L-Valine, L-Tryptophan, Acetone, Isobutyric acid, Methanol, Propylene glycol, Dimethyl sulfone.

Our CSF library includes the following compounds plus DSS:
2-Hydroxybutyrate, 2-Oxoisovalerate, 3-Hydroxyisobutyrate, Acetate, Ascorbic acid, Acetoacetate, Creatine, Dimethylamine, Citrate, Choline, Glucose, Glycerol, Formate, Glutamate, Tyrosine, Phenylalanine, Alanine, Threonine, Mannose, Isoleucine, Histidine, Lysine, Serine, Lactate, 2-Oxoglutarate, myo-Inositol, Oxalacetate, Pyruvate, Succinate, Pyroglutamate, Xanthine, Urea, 3-Hydroxybutyrate, 2-Hydroxyisovalerate, Creatinine, Glutamine, Fructose, Leucine, Methionine, 3-Hydroxyisovalerate, Isopropanol, Valine, Tryptophan, Acetone, Methanol, Propylene glycol, 1,5-Anhydrosorbitol, Dimethylsulfone.
